# Bridging the Gap across the Transition to Coparenthood: Triadic Interactions and Coparenting Representations from Pregnancy through 12 Months Postpartum

**DOI:** 10.3389/fpsyg.2017.00475

**Published:** 2017-03-31

**Authors:** Regina Kuersten-Hogan

**Affiliations:** Psychology Department, Assumption CollegeWorcester, MA, USA

**Keywords:** coparenting relationship, coparenting dynamics, triadic interactions, coparenting representations, pregnancy, transition to parenthood

## Abstract

Most family researchers agree that the coparenting relationship emerges some time during the transition to parenthood, though it is unclear whether it originates in pregnancy. Previous studies demonstrated that couples' positive representations of their future coparenting relationship and harmonious coparenting behaviors observed during prenatal triadic interactions predicted better postpartum functioning. However, previous studies did not simultaneously assess prenatal coparenting behaviors and representations as predictors of postpartum coparenting. If the coparenting relationship originates during pregnancy, these behavioral and cognitive aspects of prenatal coparenting should show associations with their postpartum counterparts. Based on family systems-, attachment-, and social-learning theory, the first aim in this study was to explore whether prenatal coparenting representations and behaviors are associated with postpartum coparenting, which would suggest that both cognitive and behavioral aspects of the coparenting relationship emerge during pregnancy. A second aim was to determine whether parental coparenting representations are consistent with concurrently observed coparenting behaviors. A sample of 55 couples expecting their first child was observed during triadic interactions during pregnancy and at 3- and 12-months postpartum. Observations were coded using the Coparenting and Family Rating System. Composite scores were formed to reflect harmonious and antagonistic coparenting behaviors. Parents' representations of harmonious and antagonistic coparenting were assessed via interviews and questionnaires during pregnancy and at 3- and 12-months postpartum. Results indicated that prenatal representations of harmonious and antagonistic coparenting were associated with and predicted unique variance in respective postpartum coparenting representations. Prenatal coparenting behaviors were also associated with coparenting behaviors observed during 3-months-play and 12-months-mealtime interactions and predicted unique variance in postpartum coparenting. Surprisingly, prenatal coparenting representations were not associated with prenatal behaviors, though representations and behaviors were associated at 3 months postpartum. Findings suggest that the coparenting relationship originates during pregnancy with prenatal coparenting representations and behaviors bridging the gap across the transition to coparenthood. Future studies should include assessments of both cognitive and behavioral facets of the prenatal coparenting relationship.

## Introduction

Couples' transition to welcoming their first child into their family involves monumental changes requiring reorganization at intrapsychic and dyadic levels. The emergence of their new parental roles changes parents' sense of identity and well-being (Cowan and Cowan, 1992), shifts gender-role attitudes and behaviors (Katz-Wise et al., 2010), and modulates attachment orientations toward partners (Simpson et al., 2003). As postulated by family systems theory, the family is an open, living, organized system whose members are engaged in ongoing relationships and interactions with one another. Morphogenic forces, which operate in families during the transition to parenthood, result in partners' interrelated psychological transformations and in changes in family structure to accommodate the new family member, roles, and relationships. The transition to parenthood thus marks a critical time period in the family life cycle for the development of the coparenting relationship, though it is not yet clear whether this relationship actually begins during pregnancy or fully emerges only after birth.

While the birth of a couple's first child is a relatively brief and clearly identifiable event, the transition to parenthood involves a process that begins long before birth and possibly even before conception. During pregnancy, partners begin to prepare for their new roles and shift to a triadic family structure by forming mental representations of their future triadic relationships with their baby (von Klitzing et al., 1999). These prenatal mental representations of life with baby seem to play an essential role in couples' transition experiences, though it is not yet known whether coparenting representations are expressed in couples' prenatal behaviors and whether both representations and behaviors are manifestations of a prenatal coparenting relationship. The present study explored couples' mental representations and coparenting behaviors across the transition to parenthood in order to determine whether the coparenting relationship originates during pregnancy. Evidence of associations between prenatal and postnatal coparenting representations and behaviors would suggest that different facets of the coparenting relationship originate during pregnancy. Based on family systems-, attachment- and social-learning theories, it is hypothesized that coparenting representations and behaviors during pregnancy essentially bridge the gap across the transition to parenthood. While some characteristics of mental representations of coparenting and prenatal coparenting behaviors have been previously investigated in separate studies, no study to date has explored cognitive as well as behavioral facets of the prenatal coparenting relationship simultaneously. The present study used a prospective, multi-method, longitudinal research design, which included both representational as well as behavioral indices of prenatal coparenting, in order to determine whether the coparenting relationship begins during pregnancy.

### The transition to coparenthood

At the most general level, coparenting involves the coordination of care-giving roles and responsibilities between two or more caregivers (McHale, 1997). Coparenting dynamics observed during triadic interactions range from harmonious, supportive coparental alliances with high levels of cooperation, coparental warmth, and coparental involvement to undermining, antagonistic coparenting characterized by high levels of coparental competition and verbal sparring and low levels of warmth and cooperation (McHale and Lindahl, 2011). In addition to these overt coparenting behaviors observable during triadic family interactions, coparenting also includes joint family management (Feinberg, 2003), division of childcare responsibilities, and inter-parental differences in childrearing philosophies (Van Egeren and Hawkins, 2004).

Minuchin's structural family theory (Minuchin, 1985) exerted a seminal influence on family researchers' conception of the coparenting relationship during the transition to parenthood. As the “executive subsystem” of the family, the emerging coparental relationship requires a major reorganization of the family structure. The addition of the child- and coparental-subsystems shifts the family unit from a dyadic to a triadic system. The question is whether the child needs to be physically present as interactive partner in triadic interactions in order for these new subsystems to emerge, or whether parents' prenatal representations of future triadic interactions are sufficient to launch the coparenting relationship during pregnancy.

Given the major structural changes families encounter during the transition to coparenthood, it is not surprising that a vast number of researchers documented the challenges associated with this transition, though mostly for the marital and parent-child relationships rather than the coparental relationship. This past research has demonstrated that the transition to parenthood seems to amplify difficulties in the pre-birth marital relationship placing some couples at risk for developing postpartum adjustment difficulties (Kluwer and Johnson, 2007). Most new parents experience declines in relationship satisfaction after birth (Lawrence et al., 2010) due to traditionalizing of partners' roles in the family, increased stress, and sleep deprivation (Medina et al., 2009), though a few couples report decreases in marital conflict after becoming parents (Holmes et al., 2013). In addition, couples' prenatal expectations of their future parent-child relationships, and especially postpartum violations of these expectations, have been linked to their postpartum adjustment (Flykt et al., 2012; Lindblom et al., 2014). While there is ample evidence that prenatal marital and parent-child subsystems forecast postpartum family dynamics, prenatal coparenting is still a relatively new area of investigation into the transition to parenthood. Since the transition to coparenthood has not yet been fully charted, it is unclear whether the coparenting relationship originates during pregnancy or begins only after the birth of partners' first child.

### Internal working models and mental representations of coparenting

A handful of studies have demonstrated that prenatal coparenting behaviors are consistent with coparenting dynamics observed in the postpartum period (Carneiro et al., 2006; Simonelli et al., 2013; Altenburger et al., 2014), though it is still unclear why prenatal family processes forecast postnatal interactions in the face of major structural changes. One way to explain the surprising continuity between prenatal and postnatal coparenting dynamics is via parents' mental representations of coparenting which form during pregnancy and perhaps even earlier. Based on attachment theory, it is hypothesized that parents' mental representations of coparenting dynamics may bridge the gap between dyadic family contexts during pregnancy and triadic family contexts in the postpartum period.

According to attachment theory (Bowlby, 1969, 1988), infants' emotional bonds formed with caregivers in the first year of life become internalized into “working models” of attachment that are subsequently utilized in forming intimate relationships with others outside of the family. These working models or representations of intimate relationships essentially shape individuals' approaches to close relationship into adulthood and are thought to explain the remarkable stability of attachment security across individuals' lifetimes as well as across relationships and even generations (Fonagy et al., 1991; Fraley, 2002; Waters et al., 2003; Shah et al., 2010).

Similar processes are hypothesized to explain the continuity between parents' prenatal representations of coparenting and postpartum coparenting representations and behaviors (von Klitzing et al., 1999; McHale et al., 2004; McHale and Rotman, 2007). Internal working models of triadic family dynamics are hypothesized to develop over the course of childhood, and subsequently shape adult partners' expectations of triadic interaction patterns and coparenting relationships during pregnancy and the postpartum period. As children are exposed to models of coparenting relationships in their families of origin, they begin to form mental representations of coparental dynamics and triadic interaction patterns, which they often enact during symbolic play during the preschool years. During the formative stages of the family life cycle when couples' dyadic relationships are established, each partner's individual representations of these family relationships formed during their childhood are activated. In contrast to parental working models of attachment, each partner brings their own individualistic coparenting representations to the newly evolving family system. Partners' differing, individualistic working models are likely to mutually influence and shape one another as they mentally shift from the family dyad to the family triad. Essentially, these internal working models of family relationships may bridge the gap between prenatal and postnatal family structures and dynamics as couples make room for the next generation. Consistent with this explanation, Van Egeren (2003) found that fathers' perceptions of more successful coparenting relationships in their families of origin predicted more positive expectations of their own coparenting relationship.

Internal working models of triadic family dynamics are conceptualized in this study as mental representations involving various aspects of family processes, including expectations of the degree of harmony vs. conflict that will be experienced in the coparental relationship. Consequently, parental representations of coparenting involve cognitive facets of the coparenting relationship such as caregivers' perceptions of the overall quality of their coparental relationship, appraisals and anticipations of their own and their partners' specific coparenting behaviors, perceived differences between partners' parenting attitudes, and partners' violated expectations of childcare responsibilities. These prenatal representations of the future coparenting relationship are hypothesized to be associated with couples' prenatal coparenting behaviors as well as with their postpartum coparenting relationship.

### Links between prenatal coparenting representations and postpartum functioning

While the majority of previous studies did not explicitly focus on nor label couples' mental representations of coparenting as such, their explorations of parents' prenatal perceptions, ideas, beliefs, and expectations surrounding the future coparenting relationship constitute aspects of these coparental representations. Mental representations of coparenting and family dynamics can be invoked in couples expecting their first child and have been found to predict families' postpartum functioning. For example, couples' realistic representations of marriage and parenthood during pregnancy (Curran et al., 2009) and their less pessimistic expectations of the future coparenting relationship (McHale and Rotman, 2007) foreshadowed more adaptive postpartum coparenting. Specifically, partners' negative expectations of their future coparenting relationship predicted less coparental cooperation and warmth during interactions with their 3-months olds, especially when babies were perceived as more difficult to soothe (McHale et al., 2004). Pregnant couples' triadic capacity, defined as their ability to fantasize about their future relationship with their child while neither excluding themselves nor their partners in these fantasies, also predicted couples' triadic interactions at 4 months postpartum as well as infants' triadic capacity (von Klitzing et al., 1999).

Partners' expectations of future childcare responsibilities constitute another important aspect of their prenatal coparenting representations, which have been found to predict their postpartum functioning. Past research has demonstrated that mothers' prenatal expectations of greater childcare responsibilities were associated with greater pessimism about the future coparenting relationship (McHale et al., 2004) and greater discrepancies between mothers' expected and actual postpartum childcare division were associated with less supportive postpartum coparenting (Khazan et al., 2008). However, as discrepancies between prenatal childcare expectations and postpartum childcare decreased over the course of early childhood, mothers' perceptions of their coparenting relationship improved (Van Egeren, 2004). It appears that especially for mothers, prenatal representations of childcare division with their partners that were later disconfirmed by mothers' actual postpartum childcare responsibilities had negative effects on the coparenting relationship.

Partners' prenatal representations also include their fantasies of their baby-to-be that shape postpartum triadic interactions. Ammaniti and Gallese (2014) noted that pregnant couples' observations of their unborn baby during 4D ultrasounds provided them with visual information about their baby's physical features and movements, activated their innate care-taking instincts, and allowed them to perceive their baby as having intentions and feelings. These prenatal representations are likely to pave the way for transforming the couple's dyadic relationship into the new family triad.

It is clear that couples' prenatal representations of coparental and family relationships predict the quality of their postpartum functioning. However, most studies to date investigated single aspects of coparenting representations rather than including a variety of different coparenting representations, such as partners' expectations for future childcare responsibilities, anticipations of coparenting support vs. undermining, and perceptions of similarities vs. differences in parenting approaches. In addition, prior research has not yet established whether prenatal coparenting representations are consistent with partners' postpartum representations. Such associations between prenatal and postnatal coparenting representations would provide evidence that at least the cognitive aspects of the coparenting relationship originate during pregnancy.

### Observations of prenatal coparenting behaviors

Parental coparenting representations involve the cognitive facets of the coparenting relationship and should theoretically be interrelated with behavioral aspects of coparenting. According to Bandura's social learning theory (Bandura, 1977), individuals' thoughts and behaviors are reciprocal determinants of one another. Peoples' expectations influence how they behave, and the outcomes of their behaviors change their expectations. Applied to coparenting during the transition to parenthood, social-learning theory implies that parental expectations of their coparental relationship will influence their coparenting behaviors. In turn, experiences partners have during interactions with coparenting partners will shape their expectations and perceptions of their future coparenting relationship and thus impact their coparenting representations. These proposed and interrelated processes provide the impetus in the present study to focus on mental representations of the coparenting system combined with their behavioral manifestations directly observed during triadic interactions. It is proposed that beliefs and expectations of future triadic interactions partners form during pregnancy guide their behaviors during prenatal interactions with one another as well as shape their postpartum coparenting relationship.

Despite the fact that cognitive and behavioral facets of the coparenting relationship are likely to be interrelated, the majority of coparenting investigations during the transition to parenthood to date avoided direct observations of prenatal coparenting behaviors. One reason for the dearth of direct observations of prenatal coparenting behaviors may be that pregnant couples lack explicit focal points for their coparenting behaviors since their unborn babies are obviously unable to partake in the interaction as active partners. In addition, there is some disagreement among family researchers regarding the interpretation of links between prenatal antecedents and postpartum coparenting. Van Egeren and Hawkins (2004) argued that prenatal perceptions of coparenting dynamics should simply be regarded as predictors of subsequent postpartum coparenting rather than as manifestations of actual prenatal coparenting. However, other researchers have uncovered links between prenatal and postnatal family alliances and coparenting behaviors, which suggest the presence of a prenatal coparenting relationship.

In order to encourage pregnant couples to enact their coparenting representations, prenatal observation tasks need to encourage the activation of triadic mindsets in partners. Fivaz-Depeursinge and colleagues (Carneiro et al., 2006) developed a unique, **prenatal observational paradigm**, the Prenatal Lausanne Trilogue Play (PLTP), which is perfectly suited to encourage couples' enactment of their prenatal coparenting representations. In this observational task validated with Swiss (Carneiro et al., 2006), Italian (Simonelli et al., 2013) and more recently also American families (Altenburger et al., 2014), expectant couples are asked to engage in triadic interactions with a doll symbolizing their baby. One of the major advantages in using the PLTP involves the fact that partners do not need to be aware of nor verbalize their mental representations of triadic family interaction patterns. Rather, their mental representations become evident in and guide their behaviors displayed during interactions with their partner and their symbolized child. The value and validity of the PLTP has been demonstrated in studies originating from different laboratories and different countries, which have all found evidence of continuity between prenatal and postnatal triadic dynamics. For example, family alliances observed during the PLTP in Swiss families were found to predict family alliance, coparental cooperation, and family warmth at 3 months after birth (Carneiro et al., 2006) and even during the toddler years (Favez et al., 2006). In addition, Altenburger et al. (2014) found that American couples who displayed higher quality coparenting during the PTLP engaged in more supportive and less undermining coparenting at 9 months postpartum after controlling for marital functioning. This study focused specifically on prenatal coparenting behaviors and provided preliminary evidence for the emergence of the coparenting relationship during pregnancy. However, Altenburger and colleagues' coding of prenatal coparenting behaviors was limited to assessing only coparental cooperation, family warmth, and structuring rather than providing a comprehensive assessment of the prenatal coparenting relationship.

In an adaption of the original PLTP, Ammaniti and Gallese (2014) replaced the doll used to symbolize the baby with video-recorded 4D ultrasound images of couples' unborn babies and asked couples to interact with these ultrasound images. They found that couples imitated fetal movements they saw in the video recordings of their unborn child and smiled at their fetuses on the screen more than they smiled at their physically present partners. The researchers interpreted these parental behaviors during pregnancy as indication that couples already felt affiliations with their new parenting roles. However, Ammaniti and Gallese did not directly assess couples' mental representations of their future parental or coparental roles.

In summary, past research has demonstrated that prenatal coparenting and family dynamics predict postpartum dynamics suggesting that behavioral aspects of the coparenting relationship originate already during pregnancy. Whether the unborn child is mentally represented in partners' minds, symbolically represented by a doll, or represented via ultrasound videos, it is clear from observations of caregivers' behaviors that their prenatal interactions are triadic in nature and constitute prenatal manifestations of the coparenting relationship. However, the current literature has not yet explored representational and behavioral aspects of prenatal coparenting simultaneously, which is an important oversight as coparenting representations and behaviors are likely to be interrelated. The exploration of links between prenatal and postnatal cognitive as well as behavioral facets of coparenting would provide even stronger support for the argument that the coparenting relationship emerges during pregnancy than previous investigations have been able to provide. In addition, the simultaneous assessment of parental representations and behaviors during pregnancy would help to determine if mental representations of coparenting are consistent with coparental behaviors observed during triadic interactions. There is some evidence that parental self-reports of postpartum coparenting are not consistent with directly observed coparenting behaviors (Karreman et al., 2008; Brown et al., 2010), though no prior study explored this issue during pregnancy. A comprehensive study of prenatal coparenting representations and behaviors would clarify how cognitive and behavioral aspects of the coparenting relationship emerge in an interrelated fashion across the transition to parenthood.

### The current study: aims and hypotheses

The first aim in this study was to determine whether parents' prenatal mental representations of coparenting and prenatal coparenting behaviors predict partners' postpartum coparenting representations and behaviors. Unlike previous investigations, the current study simultaneously explored both cognitive and behavioral facets of the prenatal coparenting relationship in order to determine whether both facets of this relationship originate during pregnancy. This also placed the current study in a unique position for investigating the relative contribution of both prenatal coparenting behaviors and prenatal coparenting representations in postpartum coparenting. Associations between multiple measures of prenatal and postpartum coparenting representations and behaviors can provide stronger evidence for the prenatal origins of the coparenting relationship than previous studies using single indices of coparenting representations and behaviors have been able to provide. Based on past research, it was predicted that both cognitive and behavioral aspects of prenatal coparenting would be associated with the respective postpartum indices. More specifically, it was hypothesized that harmonious and antagonistic coparenting behaviors observed during pregnancy would be associated with and explain unique variance in harmonious and antagonistic coparenting behaviors at 3- and 12-months postpartum. Similarly, parents' representations of harmonious and antagonistic facets of their future coparenting relationships voiced during pregnancy were hypothesized to predict and explain unique variance in representations of their coparenting relationships after birth.

The second aim in this study was to determine whether parents' coparenting representations are associated with their concurrent coparenting behaviors observed during triadic interactions from pregnancy through 12 months postpartum. In other words, the second aim in this study was to explore whether parental representations of coparenting are reflected in their directly observed coparenting dynamics starting in pregnancy. Since no previous study to date included observational and self-report measures of coparenting during pregnancy and postpartum studies on the consistency between parent-reported and observed coparenting yielded mixed results, no specific predictions were advanced for this study aim.

By utilizing a comprehensive, multi-method design to assess both cognitive and behavioral aspects of coparenting during pregnancy and at 3- and 12-months postpartum, the present study addressed some of the methodological shortcoming of previous research. Coparenting behaviors in the present study were assessed via direct observations of triadic play interactions during pregnancy as well as during play, caretaking, and mealtime interactions at 3- and 12-months postpartum. Partners' coparenting representations were assessed via interviews and questionnaires during pregnancy and at 3- and 12-months postpartum and included their perceptions of their family-of-origin coparenting, expectations of their future coparenting behaviors and childcare responsibilities, and their beliefs about parenting. Partners' postpartum coparenting representations involved their perceptions of their current postpartum coparenting relationships and childcare responsibilities.

## Methods

### Participants

A sample of 55 couples expecting their first child was studied during couples' last trimester of pregnancy and at 3- and 12- months postpartum. Sociodemographic characteristics of this sample are listed in Table 1. At 3 months postpartum, 52 of the original 55 families (5.5% attrition^1^) remained in the study (26 boys, 26 girls). At 12 months postpartum, 44 families (24 boys, 20 girls) remained in the study (15% attrition^2^).

**Table 1:** **Sociodemographic sample characteristics assessed during pregnancy**.

	**Mothers**	**Fathers**
Mean Age	31.7 (*SD* = 4.65)	33.8 (*SD* = 5.98)
**RELATIONSHIP STATUS**		
Married	53 (96.4%)	
Cohabitating	2 (3.6%)	
Mean number of years married	3.76 (*SD* = 2.96)	
Mean number of years in relationship	6.75 (*SD* = 3.43)	
**HIGHEST LEVEL OF EDUCATION**		
High School/GED	5 (9.1%)	6 (10.9%)
Associate/vocational degree	10 (18.2%)	8 (14.5%)
Bachelor's degree	19 (34.5%)	24 (43.6%)
Graduate degree	21 (38.1%)	17 (31%)
**ETHNICITY**		
White	47 (85.5%)	50 (90.9%)
Latino	3 (5.5%)	1 (1.8%)
African-American	2 (3.6%)	1 (1.8%)
Asian/Pacific Islander	3 (5.5%)	3 (5.5%)
**EMPLOYMENT STATUS**		
Not Employed	4 (7.3%)	2 (3.6%)
Employed <35 h/week	10 (18.2%)	7 (12.7%)
Employed ≥ 35 h/week	40 (72.7%)	45 (81.8%)
Fulltime Student	1 (1.8%)	1 (1.8%)
**YEARLY GROSS INCOME PER FAMILY (2008)**		
$25,001–$35,000	3 (5.5%)	
$35,001–$45,000	2 (3.6%)	
$45,001–$55,000	1 (1.8%)	
$55,001–$65,000	21 (38.1%)	
$65,001–$75,000	9 (16.4%)	
$75,001–$85,000	6 (10.9%)	
$85,001–$95,000	8 (14.5%)	
$95,001 and over	5 (9.1%)	

### Procedures

The study reported here was part of a larger, longitudinal investigation into the transition to parenthood, which included assessments of family emotional expressiveness and marital functioning and social-emotional assessments of infants at 12 months. Only measures pertaining to coparenting dynamics were included in this report. This study was carried out in accordance with the recommendations of the Internal Review Board Guidelines at Assumption College with written, informed consent from all participants. All participants gave their written, informed consent in accordance with the Declaration of Helsinki.

Couples were recruited from childbirth classes held in hospitals and a childbirth education center located in a midsized, northeastern U.S. city. Families were studied prenatally and at 12 months postpartum in a College laboratory and visited in their homes at 3 months postpartum. Families were compensated for completing each study visit.

Coparenting dynamics were directly observed during pregnancy and at 3- and 12-months postpartum during various structured and unstructured tasks. During their last trimester of pregnancy, couples' coparenting behaviors were observed using an adapted version of Carneiro et al.'s PLTP (Carneiro et al., 2006). As in the original PLTP, couples were introduced to this task with a brief interview designed to invoke mental representations of their unborn child. Then, couples were presented with a doll and asked to play as they imagined they would with their baby. The doll had detailed body parts (head, neck, torso, arms, legs, hands, feet), was similar in size and weight to a newborn baby, and dressed in gender-neutral infant clothes. Head and facial area were covered with a flesh-colored cloth (matching couples' predictions of their babies' skin tones). The examiner placed the doll into an infant seat arranged within easy reach and at equal distance from each partner emulating the triangular seating arrangement of the original PLTP. Couples were instructed to incorporate four, self-timed parts of the standardized PLTP into their play: For the first two parts, each partner was asked to individually play with “baby” while the other partner was present; for the third part, both partners were asked to play together with “baby,” and for the last part, couples were asked to pretend that their “baby” fell asleep and to discuss their experiences during this task. Toys were permitted though not encouraged. In the present sample, all couples agreed to participate in this task. The PLTP was videotaped using two cameras capturing parents' facial expressions, gestures and behaviors. The mean length of the PLTP was 7.8 min (range 2.0–17.5 min).

At 3 months postpartum, coparenting dynamics were observed in families' homes during an adapted version of the **postnatal Lausanne Trilogue Play** (LTP, Fivaz-Depeursinge and Corboz-Warnery, 1999). Family members were seated in a triangular arrangement with parents at equal distance from and facing their infant, who was placed in an infant seat. Parents were asked to incorporate four different parts in their play: Dyadic parent-infant play in the presence of the other parent, parent-parent-infant triadic play, and parental discussion of this task while infants watched. Parents were instructed to play as they normally would without taking infants out of their seats or using pacifiers. Toys were permitted though not encouraged. Interactions were videotaped using two cameras capturing infants' whole bodies and faces and parents' faces and upper bodies. The mean length of the postnatal LTP was 7.5 min (range 2.1–17.2 min).

Coparenting dynamics were also observed during a **triadic caretaking task at 3 months** in which both parents were asked to work together on changing their baby's diaper. Caretaking interactions were videotaped using one camera to capture family members' behaviors though not necessarily their facial expressions. The mean length of the caretaking task measured from the time infants were laid down for diaper changes to the time they were picked up after diaper changes was 3.1 min (range 1.3–8.5 min).

Families were again observed in the laboratory during **12-months triadic family play** consisting of free play and cleanup, teaching tasks (puzzle, picture book, and rhyming game), and a pretend picnic with replica foods and dishes. The mean length of family play was 21.5 min (range 16.1–32.1 min). Families were also observed during **12-months triadic mealtime interactions**. Parents were videotaped while eating a snack together with infants seated in a toddler seat and parents seated on either side of their infants around a child-sized table. The mean length of mealtime interactions was 10.8 min (range 4.5–16.1 min). Two eye-level cameras on opposite walls captured family members while they were seated in the center of the room; two additional ceiling-mounted cameras captured behaviors when families moved into the corners of the room.

In addition to direct observations of coparenting behaviors across three time points, parents were also asked to report on their coparenting representations during pregnancy and at 3- and 12-months postpartum. During pregnancy, couples rated their ideas about parenting, expectations of their future coparenting relationship, and the anticipated division of their childcare responsibilities. Partners were also interviewed separately regarding their family-of-origin coparenting experiences and their attitudes about their future coparenting relationships (Prenatal Coparenting Interview, McHale et al., 2004). At 3- and 12-months postpartum, parents reported on their perceptions of their current coparenting relationships and childcare responsibilities (Postnatal Coparenting Interview- Revised, Kuersten-Hogan and McHale, Unpublished Document).

### Measures

#### Triadic observations of coparenting behaviors

The quality of observed prenatal and postpartum coparenting behaviors was separately coded using adapted versions of the Coparenting and Family Rating Scale (CFRS, McHale et al., 2001). The CFRS comprises global scales measuring coparental competition, cooperation, verbal sparring, parental investment, and parent-child and coparental warmth observed during interactions. The first rating scale, “**Active Competition**,” involved the amount of competition between caregivers for control over the task (PLTP) or for their infants' attention or affection (postpartum interactions). During the PLTP, coparental competition included parental attempts to take charge of the order of play segments, length of playtime, or nature of interactions involving the doll. At 3- and 12-months postpartum, coparental competition included efforts to outdo one another to get their infant to look at, listen to, or interact with them and parental attempts to override their partner's initiatives with the infant. Competition between parents for the task or for infants' attention could be verbal or non-verbal and ranged from “Absolutely no instances of competition” (score of 1) to “Excessive jockeying for control” (score of 5).

The second scale of the CFRS, “**Active Cooperation**,” measured the degree of overt, active cooperation between parents and involved parents' level of facilitation and support for one another's parenting during triadic interactions. This scale ranged from “No cooperation” (score of 1) assigned when partners followed their own agendas during the interaction, or ignored their partner's efforts with the doll or infant to “Numerous clear instances of facilitation, pervasive atmosphere of cooperation,” (score of 5). The normative score involved benign acknowledgments between partners without creating an atmosphere of active cooperation (score of 3).

The third scale, “**Verbal Sparring**,” rated antagonistic, critical, or sarcastic remarks exchanged between partners in the context of triadic interactions and involved mild ribbing (jokes/sarcastic comments that playfully questioned the partner's competence in parenting) on the low end of the continuum (“No ribbing,” score of 1; one instance of mild ribbing, score of 2) to overtly, unambiguously critical remarks directed at the other parent (score of 5).

The fourth scale of the CFRS involved expressions of “**Coparental Warmth**,” which considered the amount of warmth, affection, and positive verbal and non-verbal exchanges between partners and ranged from “No looks or comments/no positive affect between partners/palpable sense of coldness between them” (score of 1), to a “Pervasive sense of warmth, affectionate touches, warm glances, signs of true connection with one another” (score of 5). Polite and respectful behaviors expressed between parents without true warmth were normative and assigned a score of 3.

The fifth scale, “**Parent-Child Warmth**,” was coded separately for each partner's expressions of warmth directed at their imagined baby symbolized by the doll (PLTP) or their infant (postpartum interactions) and ranged from “Complete absence of parental approval/palpable sense of coldness toward doll/ infant” (score of 1) to “Extremely expressive” (score of 7), reserved for a parent who uses touch, speech, and active eye contact to convey warmth throughout the triadic interaction with the doll or infant.

The sixth scale, “**Parental Investment**” in the task (PLTP) or with infants (postpartum interactions), was also coded separately for each parent. During the PLTP, this scale rated the extent to which parents were actively and fully engaged in the task (High investment- score of 5) vs. making no attempts to initiate play (score of 1). During the postpartum play, caretaking, and mealtime interactions, high parental investment involved parental suggestions of activities and full parental engagement with the infant (except during the play segment of the LTP in which the other partner was supposed to play with the infant) without taking breaks (score of 5). Low parental investment during postpartum interactions involved parental disengagement and consistent failure to initiate interactions with and respond to infants' bids (score of 1).

Five pairs of highly trained raters separately coded coparenting dynamics for the PLTP, postnatal LTP, 3-months caretaking interaction, 12-months triadic play, and 12-months triadic mealtime interaction. Inter-rater reliability was based on double-coding of 25% of the data. Intraclass correlations coefficients across all CFRS subscales ranged from 0.68 (*p* < 0.05) to 0.88 (*p* < 0.01).

#### Parent-reported coparenting representations

##### Coparenting scale

During pregnancy, partners were asked to separately rate their anticipated coparenting behaviors using the Prenatal Coparenting Scale (Kuersten-Hogan and McHale, Unpublished Document). At 3- and 12-months postpartum, parents were asked to separately rate their current coparenting behaviors using an adapted version of the Coparenting Scale- Revised (McHale, 1997). All three versions of the Coparenting Scale involved 21-item questionnaires adapted from McHale's original Coparenting Scale, which has been shown to have adequate internal consistency and concurrent validity (McHale et al., 2000). On the Prenatal and Postnatal Coparenting Scales, couples were asked to rate the frequency of their future or current coparenting behaviors including promotion of coparental harmony, disparagement of the partner, and coparental competition on a scale from 1 (“absolutely never”) to 7 (“almost constantly”).

Three composite scores measured parents' anticipation of future harmony-promoting behaviors, future disparagement of their partner, and future coparental competition during pregnancy, and their current harmony-promotion, disparagement, and competition at 3- and 12-months postpartum. At each of the three time points, mothers' and fathers' composite scores correlated significantly and were subsequently standardized and summed to form parental scores for harmony-promotion, disparagement, and competition during pregnancy, and at 3- and 12-months postpartum.

##### Ideas about parenting (PIA)

To assess partners' perceptions of inter-parental discrepancies in parenting ideas, Cowan and Cowan's “Ideas About Parenting” questionnaire (IAP) was used during pregnancy, which asked each partner to report on their own and their partner's opinions regarding 46 different child-rearing issues on a scale ranging from 1 (very much agree) to 9 (very much disagree). As outlined in McHale and Rotman (2007), discrepancy scores were calculated reflecting mothers' and fathers' perceived discrepancies between their own and their partners' parenting ideas. Higher scores indicated greater inter-parental perceived discrepancies in ideas about parenting. Mothers' and fathers' discrepancy scores correlated significantly (*r* = 0.33, *p* < 0.05) and were summed to form a parental discrepancy in parenting ideas score.

##### Who does what

During pregnancy, partners were asked to complete the prenatal version of the “Who Does What” questionnaire (Cowan and Cowan, 1988) asking them to rate their expected and ideal divisions of different postpartum childcare responsibilities (i.e., feeding, choosing toys for baby). At 3 months postpartum, partners rated their actual and ideal childcare responsibilities on the “Who Does What” questionnaire (Cowan and Cowan, 1988). A composite score measured the discrepancy between the expected division of responsibilities voiced by each partner during pregnancy and the actual childcare responsibilities reported by each partner at 3 months postpartum. Higher scores indicated greater violations of prenatally expected childcare responsibilities. Mothers' and fathers' perceptions of violated childcare expectations were correlated (*r* = 0.40, *p* < 0.01) and summed to form a parental childcare violation score.

##### Prenatal coparenting interview

During pregnancy, couples' expectations of their future coparenting relationship were measured using the Prenatal Coparenting Interview (McHale et al., 2004), a semi-structured interview which involves questions about coparenting experiences partners have had in their families of origin and questions about partners' perceptions of their anticipated future coparenting relationship. Specifically, partners were asked to rate their own parents' coparenting relationships and to reflect on characteristics of their family-of-origin experiences, which they would like to replicate and avoid in their own future families. Partners' were also asked about their ideas, conversations, and plans regarding the allocation and coordinating of their future parenting roles, their strengths and concerns about their own and their partners' future parenting, and their visions for ideal family life when their baby is 1 year old. Interviews were conducted separately with each partner, videotaped and transcribed for future coding using a set of question-specific as well as global scores. Only the global scores were used in the present study.

Three global scores based on McHale et al.'s coding system (McHale et al., 2004) considered partners' responses during the entire interview. The first global scale measured pessimistic views of the future coparenting relationship ranging from an “absence of negative outlook” (score of 1) to an “extremely negatively outlook” (score of 7). The other two global scales measured partners' positive outlooks on family life in general (not restricted to coparenting dynamics) ranging from “several clear examples of positive family dynamics expected in the future” (score of 7) to a “complete absence of references about positive family life” (score of 1), as well as partners' triadic propensity during pregnancy ranging from “predominantly dyadic or individual focus” (score of 1) to “well-elaborated, triadic propensity” (score of 7). Inter-rater reliability was based on double-coding of 25% of the data. Intraclass correlations coefficients across all three global scales ranged from 0.66 (*p* < 0.05) to 0.89 (*p* < 0.01). Mothers' and fathers' global scores correlated significantly (*r* = 0.38, *p* < 0.01 for pessimistic views of the future coparenting relationship, *r* = 0.31, *p* < 0.01 for positive outlook on general family life, and *r* = 0.60, *p* < 0.001 for triadic propensity) and were summed to form composite scores for prenatal coparenting pessimism, positive family views, and triadic propensity.

##### Postnatal coparenting interview-revised

At 3- and 12- months postpartum, partners were interviewed separately and asked about their perceptions regarding their current coparenting relationship during the Postnatal Coparenting Interview (adapted from McHale, 1997). Specifically, this semi-structured interview asked partners to describe their current coparenting and their own and their partners' strengths and weaknesses in parenting. Partners were also asked to compare their current coparenting experiences to their prenatal expectations of family life and to discuss any surprises they experienced in their own and their partners' parenting roles as well as in the changes they experienced in their couple relationship after birth. Interviews were conducted separately with each partner, videotaped and transcribed for future coding.

Although interview questions differed between the prenatal and postnatal versions of the Coparenting Interview, the same global scores used to measure parents' prenatal expectations of the future coparenting relationship were used to measure partners' pessimistic views of their current postpartum coparenting relationship, positive outlooks on their general family experiences, and triadic propensity. Inter-rater reliability was based on double-coding of 25% of the data. Intraclass correlations coefficients across all three global scales ranged from 0.60 (*p* < 0.05) to 0.74 (*p* < 0.01). Separate composite scores for 3- and 12-months assessments were calculated by summing mothers' and fathers' respective global scores for coparenting pessimism, positive family views, and triadic propensity.

### Data reduction

In order to reduce the number of observational measures of coparenting behaviors, composite scores for observed coparenting harmony and observed coparenting antagonism were computed based on inter-correlations between raw score measures of coparenting at each assessment point (see McHale and Rotman, 2007, for similar procedures used to create coparenting observation composite scores). The composite score for observed prenatal coparenting harmony was computed by summing the standardized raw scores for prenatal coparental cooperation and warmth, prenatal maternal and paternal investment, and prenatal mother-child and father-child warmth. A second composite score for observed prenatal coparenting antagonism was computed by summing the standardized raw scores for prenatal competition and verbal sparring. Parallel composite scores were calculated for harmonious and antagonistic coparenting behaviors observed at 3- and 12-months postpartum. Internal consistency for observed coparenting harmony and antagonism ranged from 0.61 to 0.89 across all three assessment times.

In order to reduce the number of coparenting representation measures, composite scores for parents' representations of harmonious coparenting and their representations of antagonistic coparenting were calculated for each assessment time separately. Composite scores of harmonious prenatal coparenting representations were computed by summing standardized raw scores for parents' positive views of future family life, triadic propensity, and expectations of future harmony-promoting behaviors. Equivalent measures for current coparenting representations were summed to form the 3- and 12-months postpartum coparenting harmony representation composites. A second composite score for prenatal representations was computed to reflect prenatal representations of coparenting antagonism by summing standardized scores for parents' pessimism about the future coparenting relationship, expectations of future disparagement and competition, and perceived differences in parenting ideas. Composite scores for antagonistic coparenting representations at 3- and 12-months were constructed by adding respective scores for parents' pessimism about their current coparenting relationship as well as for their perceptions of current disparagement and competition with their partners. Internal consistency scores for indices of coparenting harmony and antagonism ranged from 0.59 to 0.74 across all three assessment points^3^.

## Results

### Associations between prenatal and postpartum coparenting behaviors

Pearson Product Moment correlations with prenatal and postnatal composites scores measuring observed coparenting harmony and antagonism indicated numerous associations between prenatal and postpartum coparenting behaviors. As hypothesized, harmonious coparenting dynamics observed during the PLTP were significantly correlated with harmonious coparenting dynamics observed during 3- and 12-months triadic interactions (see Table 2). Pregnant couples who displayed more harmonious coparenting behaviors including cooperation, parental warmth, and parental investment while interacting with a doll also showed more harmonious coparenting at 3 months postpartum while they played with their actual baby and while they engaged in a caretaking task. In addition, observed coparenting antagonism during pregnancy characterized by coparental competition and verbal sparring was also correlated with antagonism observed during the LTP at 3 months postpartum. Similar associations were found between coparenting harmony and antagonism observed during the PLTP during pregnancy and triadic interactions during mealtimes at 12 months postpartum. Couples who displayed more harmonious coparenting behaviors during pregnancy also tended to show more harmonious coparenting behaviors when they were observed during 12-months mealtime interactions. Surprisingly, these same links were not found between prenatal coparenting behaviors and coparenting observed while families engaged in play interactions at 12 months.

**Table 2:** **Pearson product moment correlations (one-tailed) between coparenting composite scores for harmony and antagonism observed during pregnancy and at 3- and 12-months postpartum**.

	**Prenatal coparenting (PLTP)**	
	**Harmony**	**Antagonism**
**3M COPARENTING (LTP)**		
Harmony	0.59^***^	−0.28^†^
Antagonism	−0.38^**^	0.56^***^
**3M COPARENTING (CAREGIVING)**		
Harmony	0.42^**^	−0.02
Antagonism	−0.05	0.25^†^
**12M COPARENTING (PLAY)**		
Harmony	0.21	0.17
Antagonism	0.02	0.05
**12M COPARENTING (MEALTIMES)**		
Harmony	0.44^**^	0.07
Antagonism	−0.39^**^	0.34^*^

† p = 0.05;

* p < 0.05;

** p < 0.01;

*** *p < 0.001*.

### Associations between prenatal and postpartum coparenting representations

Associations were also found between partners' prenatal and postpartum representations of coparenting harmony and antagonism. As hypothesized, partners' prenatal representations of harmonious and antagonistic coparenting were associated with their coparenting representations at 3- and 12-months (see Table 3). Specifically, pregnant couples' representations of their harmonious future coparenting relationship characterized by positive expectations of their future family life, anticipations of harmony-promoting behaviors, and triadic propensity were associated with their harmonious postpartum coparenting representations described at 3- and 12-months. In addition, couples' prenatal representations of coparental antagonism characterized by pessimism about future coparenting, expectations of parenting differences, and anticipations of future disparagement and competition were associated with parents' antagonistic postpartum representations at 3- and 12-months.

**Table 3:** **Pearson product moment correlations (one-tailed) between composite scores for parents' representations of coparenting harmony and antagonism during pregnancy and at 3- and 12-months postpartum**.

	**Prenatal coparenting representations**	
	**Harmony**	**Antagonism**
**3M COPARENTING REPRESENTATIONS**		
Harmony	0.39^**^	−0.46^**^
Antagonism	−0.21	0.65^***^
**12M COPARENTING REPRESENTATIONS**		
Harmony	0.31^*^	−0.11
Antagonism	−0.16	0.68^***^
	**3-months coparenting representations**	
	**Harmony**	**Antagonism**
**12M COPARENTING REPRESENTATIONS**		
Harmony	0.53^***^	−0.33^*^
Antagonism	−0.48^**^	0.57^***^

* p < 0.05;

** p < 0.01;

*** *p < 0.001*.

### Associations between partners' coparenting representations and concurrent coparenting behaviors

Surprisingly, partners' prenatal representations of their future coparenting relationship were not associated with their concurrent coparenting behaviors observed during pregnancy (see Table 4). In addition, no systematic associations were found between parents' mental representations of their coparenting relationship and concurrently observed coparenting behaviors at 12 months. However, at 3 months postpartum, partners with more harmonious coparenting representations tended to show more harmonious coparenting behaviors observed during the LTP and partners' with more antagonistic coparenting representations tended to display more antagonistic coparenting behaviors during the 3-months caretaking interaction.

**Table 4:** **Pearson product moment correlations (one-tailed) between composite scores for parental coparenting representations and observed coparenting behaviors during pregnancy and at 3- and 12- months**.

**Observed coparenting behaviors**	**Coparenting representations**					
	**Pregnancy**		**3 months**		**12 months**	
	**Harmony**	**Antagonism**	**Harmony**	**Antagonism**	**Harmony**	**Antagonism**
**PREGNANCY (PLTP)**						
Obs. Harmony	0.09	−0.09	0.18	−0.17	0.24	−0.34^*^
Obs. Antagonism	−0.04	0.14	−0.10	0.16	0.03	0.29^†^
**3 MONTHS LTP**						
Obs. Harmony	0.06	−0.04	0.34^**^	−0.18	0.39^*^	−0.19
Obs. Antagonism	−0.09	0.30^*^	−0.16	0.20	−0.34^*^	0.33^*^
**3 MONTHS CARETAKING**						
Obs. Harmony	0.10	−0.18	0.35^*^	−0.34^*^	0.06	−0.24
Obs. Antagonism	0.08	0.02	−0.23	0.16	−0.06	0.06
**12 MONTHS PLAY**						
Obs. Harmony	−0.09	0.03	0.14	−0.11	0.11	−0.03
Obs. Antagonism	0.09	−0.18	−0.14	0.19	0.03	0.04
**12 MONTHS MEAL**						
Obs. Harmony	0.01	0.04	0.30^*^	−0.17	0.11	−0.22
Obs. Antagonism	0.19	−0.23	−0.22	0.30^*^	−0.06	0.11

† p = 0.05;

* p < 0.05;

** *p < 0.01*.

### Prenatal coparenting predictors of postpartum coparenting

Couples' prenatal representations of their future coparenting relationship were not associated with their subsequent postpartum coparenting behaviors observed during triadic interactions at 3- and 12-months with one exception. Partners' prenatal representations of greater antagonism were linked to greater coparenting antagonism observed during the 3-months LTP (see Table 4). Prenatal coparenting behaviors were also not associated with partners' 3-months coparenting representations, although they did show associations with partners' subsequent coparenting representations at 12 months postpartum (see Table 4).

Two sets of hierarchical regression analyses were conducted to determine whether prenatal coparenting behaviors and prenatal coparenting representations predicted unique variance in their respective postpartum coparenting measures. The first set of regression analyses explored whether coparenting behaviors observed during pregnancy explained unique variance in postpartum coparenting behaviors above variance predicted by representational measures. Prenatal representations of coparenting antagonism and 3-months representations of harmony were entered on step 1 of regression analyses. Prenatal coparenting behaviors were entered on step 2 of the regression analyses. Findings indicated that prenatal coparenting behaviors explained unique variance in postpartum coparenting behaviors above and beyond contributions made by coparenting representations (see Table 5). Specifically, prenatally observed coparenting harmony explained 32.2% of additional variance in 3-months coparenting harmony during the LTP (*p* < 0.001) and 12.3% of additional variance in coparenting harmony during caregiving (*p* < 0.05) above variance explained by parental representations of coparenting harmony at 3 months. Prenatally observed coparenting antagonism also explained an additional 25.6% (*p* < 0.001) of variance in 3-months coparenting antagonism observed during the LTP above variance predicted by prenatal representations of coparenting antagonism. With respect to coparenting harmony during 12-months mealtime interactions, prenatally observed harmony explained an additional 12.5% (*p* < 0.01) of variance above harmonious coparenting representations at 3 months.

**Table 5:** **Hierarchical regressions predicting postpartum coparenting behaviors during the 3-months LTP and caretaking task and 12-months mealtime interaction**.

	***R*^2^**	**B**	**β**
	**3-Months observed coparenting harmony (LTP)**		
Step 1: 3 months harmony representations	0.12	0.30^*^	0.24
Step 2: Prenatal observed harmony	0.44	0.58^***^	0.58
	**3-Months observed coparenting harmony (caretaking)**		
Step 1: 3 months harmony representations	0.12	0.26^†^	0.29
Step 2: Prenatal observed harmony	0.24	0.26^*^	0.35
	**3-months observed coparenting antagonism (LTP)**		
Step 1: Prenatal Antagonistic Representations	0.09	0.08	0.23
Step 2: Prenatal Observed Antagonism	0.35	0.51^***^	0.51
	**12-months observed coparenting harmony (mealtimes)**		
Step 1: 3-Months Harmony Representations	0.44	0.16	0.14
Step 2: Prenatal Observed Harmony	0.56	0.37^**^	0.41

† p = 0.06;

* p < 0.05;

** p < 0.01;

*** *p < 0.001*.

A second set of hierarchical regression analyses explored whether prenatal coparenting representations explained unique variance in postpartum coparenting representations above variance predicted by observational coparenting measures. Observations of coparenting behaviors during pregnancy and at 3 months were entered on step 1 and prenatal representations of coparenting harmony and antagonism were entered on step 2 of the regression analyses. Findings indicated that prenatal coparenting representations explained unique variance in 3-months representations of harmony and antagonism as well as in 12-months representations of antagonism above contributions made by coparenting behaviors (see Table 6). Specifically, prenatal coparenting representations of harmony explained 20.5% (*p* < 0.01) of additional variance in 3-months representations of harmony above variance explained by observed harmonious coparenting behaviors during 3-months play and caretaking interactions. Antagonistic representations during pregnancy also explained an additional 38.1% (*p* < 0.01) of variance in 3-months antagonistic representations above variance explained by 3-months observations of harmonious coparenting during play. With respect to 12-months representations of antagonistic coparenting, prenatal antagonistic representations explained an additional 39.2% (*p* < 0.001) of variance above prenatal and 3-months observations of harmonious coparenting.

**Table 6:** **Hierarchical regressions predicting parental postpartum coparenting representations at 3- and 12-months**.

	***R*^2^**	**B**	**β**
	**3-months harmony representations**		
Step 1: 3 months observed harmony (caretaking)	0.21	0.20	0.18
3 months observed harmony (play)		0.29^*^	0.35
Step 2: Prenatal harmony representations	0.41	0.47^**^	0.45
	**3-months antagonistic representations**		
Step 1: 3 months observed harmony (caretaking)	0.07	−0.12	−0.13
Step 2: Prenatal harmony representations	0.45	−0.16	−0.20
Prenatal antagonistic representations		0.37^**^	0.53
	**12-months harmony representations**		
Step 1: 3 months observed harmony (play)	0.20	0.22	0.31
3 months observed antagonism (play)		−0.60	−0.21
Step 2: Prenatal harmony representations	0.28	0.28	0.27
	**12-months antagonistic representations**		
Step 1: Prenatal observed harmony	0.13	−0.09	−0.13
3 months observed antagonism (play)		0.40	0.16
Step 2: Prenatal antagonistic representations	0.52	0.49^***^	0.64

* p < 0.05;

** p < 0.01;

*** *p < 0.001*.

## Discussion

### Prenatal origins of the coparenting relationship

This multi-measure, longitudinal investigation of triadic interactions across the transition to parenthood explored cross-time associations between prenatal and postpartum coparenting representations and behaviors in order to determine whether the coparenting relationship originates prior to birth. Findings supported the hypothesis that both cognitive and behavioral facets of the coparenting relationship emerge during pregnancy. Partners' mental representations of harmonious and antagonistic future coparenting voiced during pregnancy were associated with their mental representations of their current coparenting at 3- and 12-months and predicted unique variance in postpartum representations. These links are unlikely to be due to similarities between prenatal and postnatal measures of parental representations, as prenatal interviews and questionnaires actually asked different questions than did postpartum measures and focused on partners' future expectations rather than on their current perceptions of coparenting. While associations between prenatal and postnatal coparenting representations have not been explored in prior studies, present findings support those of other researchers who uncovered links between partners' specific representational characteristics during pregnancy and postpartum adjustment (McHale et al., 2004; Curran et al., 2009). Associations between coparenting representations before and after birth suggest that partners' cognitive constructs of their coparenting relationship begin prior to birth and remain relatively consistent across the transition to parenthood. In other words, partners' initially formed expectations and prenatal visions of the general nature of their coparenting relationship appear to shape their postpartum experiences and endure in the face of major structural changes within the family system after birth. Similar to internal working models of attachment relationships proposed by attachment theorists, it is possible that internal working models of coparenting relationships formed during each partner's childhood influence the way in which partners think about their coparenting relationship after birth. Partners' prenatal expectations of support or competition from their coparenting partner carry over into their appraisals of the ongoing coparenting relationship and color how they perceive their partners' intentions and behaviors during triadic postpartum interactions.

Findings in this study also indicated that prenatal coparenting behaviors observed while partners' engaged in triadic interactions with a doll were associated with their postpartum triadic interactions involving their actual babies. Indeed, prenatal coparenting behaviors emerged as unique predictor of postpartum coparenting above parental representations of the coparenting relationship. Links between prenatal and postpartum coparenting behaviors suggest that in addition to cognitive facets, behavioral aspects of the coparenting relationship originate in pregnancy as well. These associations between prenatal and postnatal family dynamics would be very difficult to explain from a structural family perspective (Minuchin, 1985) without accepting the notion that the coparenting relationship exists during pregnancy. According to structural and family systems theory, the newly emerging coparental and parent-child subsystems lead to major restructuring of the family system. The continuity between triadic dynamics observed during pregnancy and the end of the first postpartum year suggests that this restructuring already began to take place prior to birth. This interpretation is also in line with findings by previous coparenting and family researchers who uncovered evidence of couples' triadic capacities during pregnancy (Von Klitzing and Buergin, 2005) and the predictive value of prenatal coparenting dynamics in couples' postpartum functioning (Altenburger et al., 2014). While the prenatal coparenting relationship most likely undergoes some revision after birth and in this sense may also be regarded as an antecedent to the postpartum coparenting relationship, partners' general collaboration or undermining characteristic of their prenatal triadic interactions remains relatively stable across the transition to parenthood and suggests the emergence of this coparental relationship prior to birth.

On the other hand, any explanation of cross-time stability in coparenting behaviors needs to consider the potential influence of methodological similarities between the triadic interaction tasks used during pregnancy (PLTP) and at 3-months postpartum (LTP). The prenatal and postnatal observational contexts may have encouraged similar triadic interactions as they both structured parents' play into the same 4 segments (two dyadic and one triadic play segment plus parental discussion) and used the same triangular seating arrangement. However, the prenatal and postnatal LTP also differed in at least one important respect: The presence of the baby as active interactional partner. Almost every couple in the present study spontaneously commented on the relative ease of interacting with their actual baby in comparison to having to interact with a doll during pregnancy, which provides further evidence that task similarities are unlikely to represent the main reason for associations found between prenatal and postnatal dynamics. In addition, it is also important to note that partners' prenatal coparenting during the PLTP was associated with postpartum coparenting behaviors observed during dissimilar, unstructured postnatal interactions suggesting that the consistency between prenatal and postnatal coparenting extends beyond similarities of task characteristics. Findings in the present study expanded previous research (Carneiro et al., 2006; Favez et al., 2006; Altenburger et al., 2014) by demonstrating that prenatal coparenting behaviors observed during a structured and somewhat peculiar laboratory context are also consistent with naturalistic caretaking interactions at 3 months and naturalistic mealtime interactions at 12 months postpartum. This study therefore provides further evidence that the behavioral aspects of couples' coparenting relationship emerge prior to birth, as prenatal coparenting behaviors are linked to postpartum coparenting observed in several different family situations.

The finding of continuity between prenatal and postnatal coparenting representations and behaviors does not imply that the prenatal coparenting relationship is not subject to some revision and change during the postpartum period. Previous studies have demonstrated that infants as young as 3 months of age play active roles in shaping their family dynamics via rapid alternations of their gazes directed at each parent during triadic interactions (McHale et al., 2008; Fivaz-Depeursinge et al., 2012). It would be difficult to argue that infants' characteristics and behaviors influence prenatal triadic interactions in their families. In addition, violations of prenatal expectations of child care responsibilities and violations in the expectations of their parent-child relationship have been shown to impact partners' coparenting experiences (Van Egeren, 2004; Khazan et al., 2008; Flykt et al., 2012; Lindblom et al., 2014). Perhaps it is these very changes in the coparenting relationship after birth, which motivated a few researchers to regard parents' prenatal ideas and expectations merely as precursors or antecedents rather than as indicators of an existing coparenting relationship during pregnancy (Van Egeren, 2003). However, most previous studies failed to directly observe prenatal coparenting behaviors and those investigators who relied on prenatal observations did not study these in conjunction with also assessing parents' coparenting representations. The present study using multi-method assessments of prenatal coparenting is thus able to provide further evidence for the emergence of the coparenting relationship during pregnancy, as it demonstrates that both cognitive and behavioral aspects of prenatal coparenting provide meaningful glimpses into the postpartum coparenting relationship.

Finally, the present study's findings indicated that representational and behavioral facets of prenatal coparenting each explained unique variance in postpartum coparenting. This suggests that when partners enact triadic play with their unborn child during pregnancy or contemplate their future coparenting dynamics, their prenatal coparenting behaviors and representations forecast the postpartum coparenting relationship better than either their representations or behaviors are able to predict on their own. These findings highlight the importance of including both cognitive and behavioral measures of prenatal coparenting in studies designed to predict the postpartum coparenting relationship.

### Interrelationship between cognitive and behavioral aspects of coparenting

The second aim in the present study was to explore the interrelationships between partners' coparenting representations and their concurrent coparenting behaviors during pregnancy. Social-learning theory would predict that cognitive and behavioral facets of the coparenting relationship are interrelated during pregnancy and the postpartum period. However, the present study did not find parental coparenting representations during pregnancy to be associated with their concurrent coparenting behaviors, nor were these links uncovered at 12 months postpartum. In other words, parental representations of their future coparenting relationship were not reflected in partners' directly observed coparenting dynamics during pregnancy. In addition, parental representations were also not associated with observations of parental coparenting behavior at 12 months postpartum, though they were associated at 3 months postpartum. At first glance, these findings do not appear to support predictions based on social-learning theory. Reasons for the lack of associations between coparenting representations and behaviors during pregnancy and at 12 months postpartum are likely to differ but do not necessarily imply that cognitive and behavioral aspects of the coparenting relationship do not mutually influence each other.

During pregnancy, the various facets of the coparenting relationship may still be fragmented as the coparenting relationship is just emerging. An alignment of belief structures about coparenting relationships with actual coparenting experiences requires self-observation of and reflection on coparenting interactions. In order to notice discrepancies between their representations and actual behaviors, partners require repeated and ongoing practice with triadic coparenting interactions, which may be rare during pregnancy. Other than during specific and unique situations such as the PLTP, which invoke triadic representations of future family life, couples may have few opportunities to enact their prenatal coparenting representations in everyday life. This does not mean that when these opportunities to engage in prenatal triadic interactions are provided to partners, their prenatal behaviors are meaningless with respect to their future coparenting relationship. As the present study demonstrated, prenatal coparenting behaviors predict postpartum coparenting behaviors even when they may be rarely practiced during everyday life before birth. However, without regular opportunities to observe their own and their partners' behaviors during triadic interactions during pregnancy, there are no prompts for partners to adjust their preconceived coparenting representations in order to create a better match between their representations and actual coparenting experiences. This could explain why coparenting cognitions and behaviors may initially constitute separate facets of the coparenting relationship during pregnancy that only after birth become more integrated into a solidified postpartum coparenting relationship.

It is also possible that partners are simply not yet attuned to their own coparenting behaviors or that they are not able to verbalize and report on their coparenting representations during pregnancy as well as they are at 3 months postpartum. In their longitudinal investigation of the transition to parenthood, Cowan and Cowan (1992) found that partners' sense of parental identity constituted only a small percentage of their whole identities during pregnancy, especially for expecting fathers. Responses to the Prenatal Coparenting Interview in the present study confirmed that the majority of fathers and mothers spent relatively little time thinking about and discussing specifics of their future triadic interactions and coparental roles with one another. It may be difficult for couples to have insights into or verbalize their coparenting representations if they do not yet view themselves as parents and coparental partners during pregnancy. This makes it even more vital that coparenting researchers do not solely rely on couples' verbal reports of their anticipated coparenting relationship but also observe their triadic interactions during pregnancy.

Another reason for the lack of associations between parental coparenting representations and behaviors during pregnancy is the fact that coparenting representations solicited during pregnancy focused on partners' future coparenting relationship, while prenatal observations provided insights into partners' current coparenting dynamics. One might argue that partners' representations of their future coparenting relationship constituted wishful thinking or idealistic hopes of future coparenting, while observations of their prenatal coparenting behaviors reflected their actual coparenting schemas based on their family-of-origin experiences. However, partners' expressions of pessimism about their future coparenting relationship and reports of anticipated disparagement and future coparental competition do not support this argument. Still, it is possible that partners' representations of their current prenatal coparenting relationship would have shown greater consistency with their actual behaviors observed during triadic prenatal interactions than their representations of their future coparenting relationship did.

At 12 months postpartum, reasons for the lack of consistency found between coparenting representations and observed coparenting behaviors are likely to extend beyond parents' limited insights into and awareness of their coparenting relationship. By the end of the first year of their infants' lives, parents had plenty of practice fulfilling their coparental roles and observing their own and their partner's harmonious as well as antagonistic coparenting behaviors to help them reflect on and adjust their coparenting representations. However, during this later postpartum period, triadic interactions are also more heavily influenced by infants' active participation. At 12 months postpartum, child characteristics such as their gender and temperament as well as their newly developed mobility and verbal skills may all contribute to a mismatch between parental representations and concurrently observed coparenting interactions. Bandura's social-learning theory does not imply that behaviors and cognitions have to be consistent at a specific point in time, nor that individuals' private cognitions have to match behavioral observations made by others. Behavioral and cognitive facets of the coparenting relationship may still mutually influence each other even when they do not show associations with one another at a specific point in time. With all of the developmental accomplishments and challenges 1-year-olds present to their families, parental constructs of their coparenting relationship may lag behind their current and ever-changing family dynamics. In other words, experiences with triadic coparenting interactions at 12 months may not yet have sparked revisions of parents' mental representations of these experiences, which is why parental representations at 12 months are not associated with their concurrently observed coparenting behaviors.

Aside from the aforementioned reasons, the lack of associations between representational and behavioral measures of coparenting during pregnancy and at 12 months postpartum may also be explained with the way in which parents' mental representations were measured and coded in the present study. Following in the footsteps of prior coparenting researchers, the present study coded parental representations reflected in interview and questionnaire measures for the content of parental beliefs, perceptions, and expectations. In contrast, attachment studies generally assess adults' attachment orientations by analyzing the manner in which they portray their past and current attachment relationships rather than the content of their narratives. The complexity of coparenting representations is unlikely to be captured by merely focusing on the content of parents' responses, though this is an issue that has not yet received any attention from coparenting researchers. Perhaps it is the parents' narrative coherence or their emotional integrity displayed during their descriptions of their future coparenting ideas rather than the content of their contemplations which is associated with concurrently observed coparenting dynamics during pregnancy. Future studies should explore new ways of coding parental representations of coparenting borrowing from the work of attachment researchers to capture not only what couples say about their future coparenting but also the manner in which they portray their expectations.

Of note is that in contrast to the prenatal and 12-months postpartum periods, the present study found that coparental representations were reflective of coparenting behaviors observed during 3-months play and caretaking interactions. Parents with more harmonious coparenting constructs concurrently displayed more harmonious coparenting behaviors during triadic interactions with their 3-months-olds. Parental representations at 3 months were also consistent with subsequent coparenting behaviors observed during mealtime interactions at 12 months, and parental coparenting observed during 3-months-triadic play was consistent with subsequent representations at 12 months. While the correlational design in this study does not allow for causal conclusions, it is clear that at 3-months postpartum, cognitive and behavioral features of the coparenting relationship are more closely linked than they are during earlier or later times in development. What about this stage of development in the coparenting relationship explains this better integration between cognitive and behavioral facets of the coparenting relationship? Perhaps this solidification of the coparenting relationship has to do with a return to a new homeostasis after the initial structural changes families experienced right after the birth of their first child (Minuchin, 1985). At 3 months postpartum, most non-clinical families have adapted to the introduction of their new parent-child and coparental subsystems. Coparenting partners had some time to adjust to their new roles and family life has shifted into a new routine. This settling-in phase after a major transition in family life may explain why parental representations of their coparenting relationship at this stage are in-synch with their coparenting behaviors observed during triadic interactions. At this stage of development, parents have had a chance to adjust their representations of family life to make them more consistent with their actual experiences of ongoing triadic interactions. It is clear that more research is needed to fully explore developmental changes postulated to occur in the coparenting relationship after its initial inception during pregnancy. In other words, future studies should confirm whether the coparenting relationship does indeed emerge first in more fragmented fashion during pregnancy, solidifies by 3 months postpartum, and again undergoes major revisions by the end of the first year to accommodate infants' increasing developmental advances.

### Influence of contextual factors on coparenting dynamics

In addition to exploring cognitive and behavioral facets of the coparenting relationship simultaneously at each assessment point, another contribution of this study was its inclusion of different contexts for triadic observations of postpartum coparenting. The vast majority of previous studies limited their observations of triadic interactions to play contexts, while the present study also included observations of naturalistic caretaking and mealtime situations and uncovered some interesting contextual influences on coparenting behaviors. Present findings indicated that prenatal coparenting behaviors were more predictive of postpartum coparenting dynamics observed during the 3-months LTP and 12-months mealtime interactions than they were of coparenting observed during the 3-months caretaking task or 12-months triadic play interactions. Perhaps family and coparenting dynamics change to some extent depending on whether families are engaged in daily, routine activities vs. enjoying themselves during play interactions. On the other hand, coparenting dynamics are also likely to retain some of their characteristics across a variety of different family contexts. Context-dependent fluctuations in coparenting dynamics may provide yet another explanation for not finding associations between parental coparenting representations and observations of coparenting behaviors. While representations are formed and adjusted based on parents' reflections on their cumulative experiences across a variety of different everyday situations, their coparenting behaviors are observed during a relatively brief, single, triadic interaction task. This raises important concerns for the choice of triadic tasks used in observational studies of coparenting, though unfortunately the extent to which coparenting behaviors are context-dependent or remain stable across different family life situations has not yet received much attention from coparenting researchers.

Another methodological improvement in the present study over previous research was the inclusion of both cognitive and behavioral measures in the exploration of the coparenting relationship across the transition to parenthood. Assessments of parents' prenatal representations can provide insights into their perceptions and mental schemas about future family roles and relationships that cannot be directly observed and which are likely to contribute to new parents' experiences in the early postpartum period. In contrast, direct observations of triadic interactions during pregnancy tap into aspects of the coparenting relationship, which lie outside the realm of couples' conscious awareness. As this study indicated, parental coparenting representations and behaviors during pregnancy constitute different facets of the emerging coparenting relationship that need to be assessed simultaneously in order to fully understand how the coparenting relationship changes across the transition to parenthood.

### Study limitations

Several limitations of this study need to be acknowledged. First, findings in the present study should be interpreted with caution due to the vast number of analyses conducted and the relatively small sample size. Future studies should replicate current findings using larger samples, which would allow for statistical analyses of the various prenatal characteristics of the coparenting relationship and their direct and indirect pathways to postpartum coparenting. Another limitation is that this study assessed the prenatal coparenting relationship within a relatively homogeneous sample consisting of predominantly White, well-educated, middle-class, two-parent families. The small sample size in the present study did not allow for an exploration of couples' demographic variables such as education, age, or socioeconomic status and their influence on the emerging coparenting relationship. Future studies need to replicate these findings with samples drawn from more diverse and at-risk populations such as pregnant teenage couples who are likely to be at risk for postpartum adjustment difficulties. Another limitation in this study was that coparenting behaviors during pregnancy were observed during only one triadic interaction context, the PLTP. As observational contexts were found to play a role in postpartum coparenting, future studies should expand the repertoire of observational contexts during pregnancy, for example by observing pregnant couples' triadic interactions during ongoing ultrasound imaging of their unborn children. Finally, couples' coparenting representations assessed during pregnancy focused on their future rather than on their current coparenting relationship, which may have obscured consistencies between partners' prenatal coparenting representations and their prenatal behaviors.

### Conclusions and suggestions for future research

In conclusion, the present study provided support for the claim that the coparenting relationship originates during pregnancy. Both cognitive and behavioral facets of the prenatal coparenting relationship provided meaningful glimpses into postpartum coparenting and suggest that partners' coparenting behaviors and representations should be regarded as manifestations of their prenatal coparenting relationship. This study contributed to the existing family literature by demonstrating how mental representations of coparenting as well as triadic interactions during pregnancy can bridge the gap to the postpartum coparenting relationship. The present study also extended Stern's model of the interdependency between parental and infant representations and interactions (Stern, 1995). Though focusing more on the mother-infant relationship, Stern argued that this family subsystem should be the target of therapeutic interventions and that parental representations are an important source of change in the parent-child relationship. The present study focused on a different family subsystem, namely the coparental relationship, but similarly emphasized the interplay of parental representation and action.

Findings in the present study have implications for interventions designed to help couples at risk for adjustment problems during the transition to parenthood. Since there is evidence that the coparenting relationship emerges prior to birth and sets the stage for postpartum coparenting, relationships high in coparental antagonism and low in harmony should already be targeted during pregnancy. Several researchers have begun to design and test such prenatal interventions for high-risk couples, which have included successful interventions for improving the coparenting relationship (see Feinberg et al., 2010). Findings in the present study suggest that these prenatal interventions should move beyond simply educating couples about challenges during the transition to parenthood and directly target partners' actual prenatal dynamics, as couples have limited insights into their own coparenting behaviors during pregnancy.

The present study involved a more comprehensive investigation of the prenatal coparenting relationship than previous studies have been able to provide and described the coparenting foundation laid during pregnancy. Future studies should trace the developmental changes the prenatal coparenting relationship is likely to experience during the transition to parenthood. While the present study included many different coparenting measures, future research should further expand the scope of exploration to include factors such as parental personality characteristics, mental health, and attachment orientations, which may influence the coparenting relationship across the transition to parenthood. Interlinked with parental characteristics are child characteristics (gender, temperament, attachment security, and triadic capacity), couple relationship factors, as well as family supports and stressors, all of which form a complex network of pathways that shape coparenting representations and behaviors during pregnancy and beyond (see Feinberg's ecological model of coparenting, 2003). Future studies with larger and more diverse samples should explore these coparenting correlates, mediators, and moderators in order to construct a comprehensive model of the developmental trajectory of the coparenting relationship across the transition to parenthood.

## Ethics statement

Internal Review Board, Assumption College. This study was carried out in accordance with the recommendations of the Internal Review Board Guidelines at Assumption College with written, informed consent from all participants. All participants gave their written, informed consent in accordance with the Declaration of Helsinki prior to any data collection. Participants were given a chance to have their questions answered about this research study prior to being asked to sign the consent forms. Participants were informed of their rights to terminate their participation at any point and for any reason without penalty. Participants were given a copy of the consent form for their records. During postpartum study visits, parents gave consent for their infants' participation in this study. Infants were only observed in the presence of their parents.

## Author contributions

RK held the sole responsibility for developing this research project, collecting, analyzing, and interpreting the data, and training and supervising research assistants who coded the data. RK also drafted and revised this manuscript, made the final approval of the version submitted for publication, and agrees to be accountable for all aspects of the work in ensuring that questions related to the accuracy and integrity of any part of the work are appropriately investigated and resolved.

### Conflict of interest statement

The author declares that the research was conducted in the absence of any commercial or financial relationships that could be construed as a potential conflict of interest. The handling Editor declared a past co-authorship with the author RK and states that the process nevertheless met the standards of a fair and objective review

## Footnotes

^1^Two families lost their babies due to unforeseen complications during birth and one family dropped out due to partners' separation. These participants' prenatal data was not included in any of the analyses.

^2^Four families moved out of state, and four families stated they were too busy to continue with the study. Families who discontinued the study prior to the 12-months assessment did not differ significantly from families who remained in the study in any of the coparenting measures.

^3^Only one summary score, namely that for 3-months antagonistic representations, showed marginal internal consistency.
